# Users' Perceptions and Trust in AI in Direct-to-Consumer mHealth: Qualitative Interview Study

**DOI:** 10.2196/64715

**Published:** 2025-05-20

**Authors:** Katie Ryan, Justin Hogg, Max Kasun, Jane Paik Kim

**Affiliations:** 1 Department of Psychiatry and Behavioral Sciences Stanford University School of Medicine Stanford University Palo Alto, CA United States

**Keywords:** artificial intelligence, mobile health, mHealth, trust, ethics, end users, qualitative study, semistructured interviews

## Abstract

**Background:**

The increasing use of direct-to-consumer artificial intelligence (AI)–enabled mobile health (AI-mHealth) apps presents an opportunity for more effective health management and monitoring and expanded mobile health (mHealth) capabilities. However, AI’s early developmental stage has prompted concerns related to trust, privacy, informed consent, and bias, among others. While some of these concerns have been explored in early stakeholder research related to AI-mHealth, the broader landscape of considerations that hold ethical significance to users remains underexplored.

**Objective:**

Our aim was to document and explore the perspectives of individuals who reported previous experience using mHealth apps and their attitudes and ethically salient considerations regarding direct-to-consumer AI-mHealth apps.

**Methods:**

As part of a larger study, we conducted semistructured interviews via Zoom with self-reported users of mHealth apps (N=21). Interviews consisted of a series of open-ended questions concerning participants’ experiences, attitudes, and values relating to AI-mHealth apps and were conducted until topic saturation was reached. We collaboratively reviewed the interview transcripts and developed a codebook consisting of 37 codes describing recurring or otherwise noteworthy sentiments that inductively arose from the data. A single coder coded all transcripts, and the entire team contributed to conventional qualitative analysis.

**Results:**

Our qualitative analysis yielded 3 major categories and 9 subcategories encompassing participants’ perspectives. Participants described attitudes toward the impact of AI-mHealth on users’ health and personal data (ie, influences on health awareness and management, value for mental vs physical health use cases, and the inevitability of data sharing), influences on their trust in AI-mHealth (ie, endorsements and guidance from health professionals or health or regulatory organizations, attitudes toward technology companies, and reasonable but not necessarily explainable output), and their preferences relating to the amount and type of information that is shared by AI-mHealth apps (ie, the types of data that are collected, future uses of user data, and the accessibility of information).

**Conclusions:**

This paper provides additional context relating to a number of concerns previously posited or identified in the AI-mHealth literature, including trust, explainability, and information sharing, and revealed additional considerations that have not been previously documented, that is, users’ differentiation between the value of AI-mHealth for physical and mental health use cases and their willingness to extend empathy to nonexplainable AI. To the best of our knowledge, this study is the first to apply an open-ended, qualitative descriptive approach to explore the perspectives of end users of direct-to-consumer AI-mHealth apps.

## Introduction

### Background

Direct-to-consumer mobile health (mHealth) apps present an opportunity for widely accessible health management and monitoring, with the integration of artificial intelligence (AI) offering immense promise to enhance the effectiveness and expand the capabilities of these apps [1]. Indeed, many direct-to-consumer AI-enabled mHealth (AI-mHealth) apps are already in widespread use by individuals seeking to address particular health concerns, obtain personalized insights into their health, promote health-seeking behaviors, and help set and achieve well-being goals [2].

While AI-mHealth apps offer potential for improving proactive health management and monitoring, the rapid pace of AI innovation has outpaced research efforts aiming to facilitate the ethical development and adoption of AI-based apps and maximize their benefits. Recent normative work has articulated concerns related to privacy, informed consent, and bias, among others [3-5]. For example, integral to the training of AI models and the appeal of AI use cases (eg, personalized health recommendations and digital phenotyping based on ecological momentary assessments) is the collection of behavioral data, many times more than what are typically analyzed by mHealth apps that do not use AI, and these data may encompass a wider range of tracking modalities [6,7]. Mobile devices’ capabilities to passively collect biometric data (eg, motion, heart rate, and sleep) and contextual data (eg, location and browser activity) have raised novel considerations around data privacy, including users’ ownership of these data, consent for data collection, AI capabilities of inferring private information, and potential for surveillance [8-10]. Another challenge for AI-mHealth is unwanted algorithmic bias, which, if inadequately addressed, could perpetuate existing health inequities and even widen health disparities, conflicting with the goals of personalized medicine [11-13]. A further set of considerations relates to how AI use cases may shape how users think and feel about their health status and health behaviors, for example, whether self-monitoring features, depending on how they are framed and implemented, could support or threaten experiences of autonomy and agency [6,14].

These considerations relating to trust, privacy, informed consent, bias, and autonomy have been repeatedly highlighted in the AI-mHealth literature; however, the perspectives on these issues of the stakeholders in direct-to-consumer AI-mHealth who arguably stand to assume most of its benefits and risks (ie, end users) are yet to be adequately explored in the empirical literature [15]. Empirical work assessing trust in *health care provider*-to-consumer conversational agents has shown that trust has interpersonal (eg, trust in companies and health care providers), social (eg, testimonies from loved ones and health care providers), and technological dimensions (eg, trust in the capabilities of the tool and design cues) [16]. A scoping review of studies assessing user views of AI-mHealth apps identified a number of likely barriers to adoption related to user-centered explainability, trust, empathy, usability, privacy, AI accountability, and diversity of model training and test populations [17]. Importantly, the study revealed that direct-to-consumer AI-mHealth user research has been largely limited to quantitative studies that evaluated specific dimensions of user experience collected via structured questionnaires and performance metrics generated by the apps, which tend to rely heavily on researchers’ a priori assumptions about what the major user considerations are.

The small body of published qualitative work has used interviews of actual or anticipated users of direct-to-consumer AI-mHealth apps to shed light on their attitudes and perspectives, yielding several ethically salient insights. An interview study of university students by Nadarzynski et al [18] explored their attitudes toward hypothetical conversational AI apps. The study identified several sources of hesitancy related to AI interactions (a lack of empathy and understanding, especially in mental health use cases) and accuracy and quality concerns as well as perceived advantages around anonymity, convenience, accessibility, and guidance toward relevant health services. In semistructured focus groups with young people about their attitudes toward AI-mHealth apps, Götzl et al [19] found nuanced attitudes toward data sharing and safety; participants reported willingness to share their data provided that they would truly support their health interests and expected transparency about how their data were being used, discretion in requesting more sensitive data, and user controls for personalization features. In a report by Tsai et al [20], focusing on users’ needs related to explainability in AI-enabled symptom-checking tools, interviewees reported that they often felt confused by the order and content of the differential diagnosis questions asked by these apps and hesitated to trust the accuracy of their results due to low transparency about their reasoning procedures and underlying data.

### Objectives

As AI advances, direct-to-consumer AI-mHealth apps may create more opportunities to improve population-wide health awareness, disease prevention, and well-being. To realize these aspirations, it is essential to engage with the end users whom the tools are intended to help and develop a comprehensive picture of the views of the end users of direct-to-consumer AI-mHealth apps, including their own reservations or anxieties that may remain overlooked and pose barriers to establishing trust and fostering adoption. Although previous qualitative study designs have focused on exploring users’ views on specific use cases and topics of ethical importance, no single study has examined their ethical perspectives on AI-mHealth broadly. Therefore, in this paper, we set out to describe, using semistructured interviews and a qualitative descriptive approach, the topology of ethical considerations of individuals who have experience interacting with mHealth apps with respect to AI-mHealth.

## Methods

### Study Design

The findings presented in this paper were collected and analyzed as part of a larger study that aimed to identify and describe ethically laden sentiments and anticipated issues of AI well-being apps’ use in work environments from the perspectives of current and potential future end users. This study addressed a supplemental aim to an ongoing study about the ethics of AI use in medicine (NCATS R01-TR-003505) [21]. To accomplish this aim, we conducted semistructured interviews with existing users of AI-mHealth apps (N=21) to identify and describe their perspectives relating to ethical dimensions associated with the use of such apps, specifically in the workplace.

A qualitative descriptive approach was applied to the study design, as this approach emphasizes the description and analysis of stakeholders’ experiences and perceptions using language and ideas that emerge directly from the stakeholders themselves [22,23]. While other methods of qualitative research and analysis prioritize the development or advancement of theory, qualitative description allows for the emergence of new knowledge that may not be readily available from theoretical deduction and is grounded in the experiences of participants.

### Recruitment and Participants

Adults aged ≥18 years who reported current or previous use of an mHealth app were eligible to participate in this study. Participants were recruited for this study via an electronic advertisement posted to the Stanford Psychiatry Department’s *Currently Recruiting Studies* web page. The advertisement described the study and contained a link to a web-based screening survey and was posted from August 1, 2022, through August 29, 2022.

A total of 32 individuals completed the screening survey; 31 (97%) individuals met the eligibility criteria for participation. All eligible individuals were contacted via email to schedule a Zoom (Zoom Communications, Inc) interview; 21 (68%) of these 31 eligible individuals scheduled and completed an interview. Interviews continued until the minimum target sample size of 20 was exceeded. At that point, interviewers jointly determined that content saturation had been reached, as no new substantive themes were emerging from additional data collection [24].

### Procedures

Semistructured interviews were used to facilitate discussion that encouraged participants to reflect on their experiences and opinions and allow them the flexibility to focus on topics that they found the most important. This approach allowed interviewers to ask unscripted follow-up questions where relevant or skip questions that did not apply in the context of the conversation. By conducting interviews via Zoom, participants outside of the local area were able to participate in the study.

**Figure 1:**
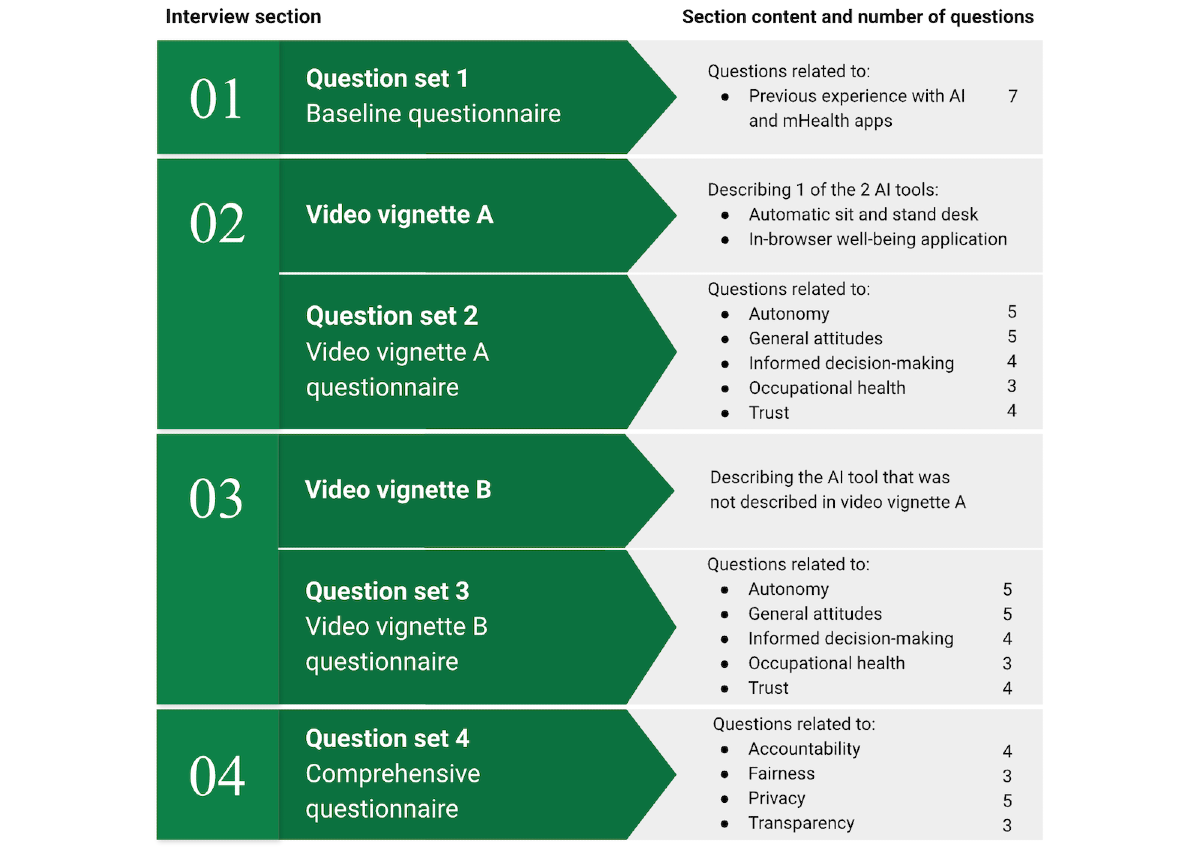
Outline of the semistructured interviews. AI: artificial intelligence; mHealth: mobile health.

The semistructured interviews contained 4 sections: a baseline question set, 2 video vignettes and associated question sets, and a comprehensive question set (Figure 1). In order to stimulate discussion of ethical considerations among a population that may not have encountered AI use cases in mHealth previously, 2 video vignettes demonstrating the use of example AI-enabled workplace health interventions were used. The presentation of these video vignettes was followed by ethically salient questions related to the presented technology (eg, questions relating to understanding, trust, and autonomous decision-making).

Interviews were conducted between August 2022 and September 2022 by 1 of our team’s 3 trained interviewers. The interviews lasted 75 minutes and 35 seconds on average (SD 24 minutes, 28 seconds; range 35-131 minutes) and were audio recorded for the purposes of transcribing.

### Data Coding and Analysis

Auto-transcriptions created by Zoom were reviewed, edited, and deidentified by a member of the research team. Data analysis was guided by the principles of qualitative content analysis [25]. This inductive approach, in which transcribed data are broken down into descriptive units that are named and sorted based on their content, allows for the emergence of codes and themes directly from the dataset [26]. As we were primarily interested in identifying and describing ethical considerations of AI-mHealth users that have been underexplored in the existing literature, this inductive approach was selected over a deductive approach, which would have relied more heavily on a priori assumptions about user priorities and perspectives.

An initial round of open coding was performed on each transcript by 2 authors. The authors reviewed their assigned transcripts independently, identified interview content that they felt was substantive, and suggested descriptive codes for this content. The authors then met as a group to discuss the descriptive codes that emerged during open coding and compare the content associated with each code. They collaboratively identified topics that recurred throughout the transcripts and established preliminary code names and definitions for these topics. The transcripts were then rereviewed by the same 2 authors using the preliminary codes and definitions as a guide. At the completion of this phase of intermediate coding, the authors met as a group to compare the coded units, further refine the code names and definitions, and draft the final version of the codebook.

The final version of the codebook contained 37 codes derived directly from the content of the interviews describing baseline characteristics and tool-specific, personal, and contextual factors influencing participant attitudes toward AI-mHealth. The transcripts and codebook were then uploaded to NVivo (version 1.0; Lumivero) for final coding, which was completed by a single research team member and then reviewed for consistency by a different team member.

Data analysis began upon the completion of coding. A conventional qualitative analysis approach was used to guide the analysis [25,26]. At the completion of final coding, all authors met as a group to analyze the content and meaning of the coded units and develop categories and themes that described the associations between units. This process resulted in the identification of 3 “buckets” detailing different aspects of participants’ experiences and attitudes toward well-being AI (Table 1). For the purpose of providing an accurate and in-depth analysis of the content of these interviews, this paper will only address the codes, categories, and themes associated with bucket 1. Those associated with buckets 2 and 3 will be analyzed in future publications.

**Table 1:** Inductive coding groups and codes describing users’ perspectives toward well-being artificial intelligence (AI; N=21).

The codes used in the development of the bucket			Interviews cited, n (%)
**Bucket 1: user attitudes toward AI-enabled mHealth^a^**			
	Data collection	21 (100)	
	Data use and sharing	21 (100)	
	Availability of information	20 (95)	
	Experience with mHealth apps	20 (95)	
	Explainability of the model	20 (95)	
	Regulation	20 (95)	
	Research participation	20 (95)	
	Potential benefits	19 (90)	
	Accountability	18 (86)	
	Attitudes toward AI	17 (81)	
	Potential risks	17 (81)	
	Trust in institutions or entities	16 (76)	
	Understanding of and familiarity with AI	13 (62)	
	Potential of AI	12 (57)	
	Value	11 (52)	
	Perceived ubiquity of technology	9 (43)	
	Financial or economic factors	8 (38)	
	Influence on the mind or behavior	3 (14)	
**Bucket 2: ethical concerns relating to well-being AI in workplace settings**			
	Workplace-related factors	21 (100)	
	Trust in the tool	20 (95)	
	Bias	18 (86)	
	Data security and privacy	18 (86)	
	Autonomy and agency	16 (76)	
	Social identity and stigma	15 (71)	
	Trust in intentions	10 (48)	
	Personal health	9 (43)	
	Personal qualities or traits	9 (43)	
	Life stage	7 (33)	
	Social or historical context	6 (29)	
	Accessibility	3 (14)	
**Bucket 3: user attitudes toward features of well-being AI tools in workplace settings**			
	Degree of user’s control	21 (100)	
	Intrusiveness	19 (90)	
	Personal context	18 (86)	
	User experience	18 (86)	
	Optimization	17 (81)	
	Type of intervention	17 (81)	
	Physical context	3 (14)	

^a^mHealth: mobile health.

### Ethical Considerations

This study obtained human participant research approval from the institutional review board of Stanford University on June 21, 2022 (58118). A copy of the institutional review board–approved informed consent form was emailed to all potential participants before the interview. Interviewers then reviewed and explained the content of the consent form to all participants at the start of the interview using the screen share function on Zoom. All participants signed an electronic consent form before the start of the study-related procedures. Participants were able to opt out at any point during the consent process or during the research interview. Identifiers were removed from the dataset during the transcription process, before analysis. Participants were compensated in the form of a US $50 electronic gift card.

## Results

### Overview

The demographic information of participants can be found in Table 2. The final cohort of participants included young adults, aged between 20 and 36 years, and was primarily composed of individuals whose reported sex was female (15/21, 71%) and whose reported race was Asian (8/21, 38%), Black (5/21, 24%), or multiracial (4/21, 19%). In their open-ended responses, participants reported having experience using AI-mHealth apps, such as Apple Health, Headspace, Calm, and Flo.

**Figure 2:**
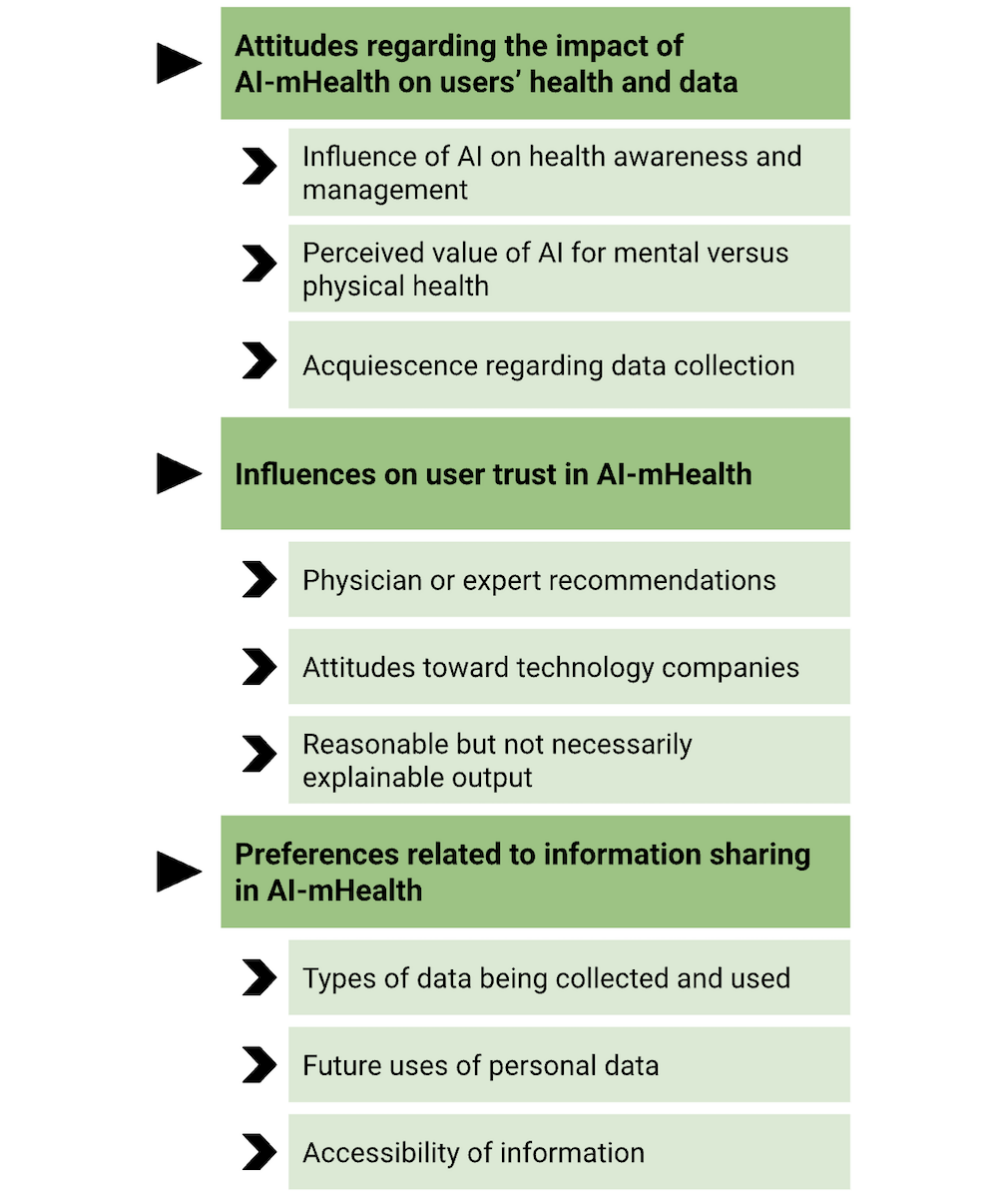
Categories and subcategories describing users’ attitudes toward artificial intelligence (AI)–enabled mobile health (AI-mHealth) apps.

All participants in this study commented on considerations related to bucket 1: user attitudes toward and understanding of AI-mHealth (Table 2). These considerations primarily arose from responses that emerged in parts 1 and 4 of the interview question set (ie, the baseline questions at the start of the interview and the comprehensive questions at the end of the interview; Figure 1). In discussions relating to attitudes and understanding of AI-mHealth, 3 major categories, each with 3 related subcategories, emerged (Figure 2). The content of these categories and subcategories is described in detail in the subsequent sections.

**Table 2:** Participant demographics (N=21).

Demographics			Values
**Age (y)**			
	Value, mean (SD)	26.8 (4.98)	
	Value, median (range)	26.0 (20.0-36.0)	
	Missing, n (%)	2 (10)	
**Sex, n (%)**			
	Female	15 (71)	
	Male	6 (29)	
**Race, n (%)**			
	Asian	8 (38)	
	Black or African American	5 (24)	
	White	3 (14)	
	Multiracial	4 (19)	
	Other	1 (5)	
**Ethnicity, n (%)**			
	Hispanic or Latino	1 (5)	
	Not Hispanic or Latino	20 (95)	
**Education^a^, n (%)**			
	High school degree or equivalent	1 (5)	
	Some college; no degree	5 (24)	
	Bachelor’s degree	6 (29)	
	Master’s degree	4 (19)	
	Doctorate	1 (5)	
	Associate, technical, or vocational degree	3 (14)	
	Missing	1 (5)	
**Employment**			
	Employed full time (≥35 h per wk)	10 (48)	
	Employed part time (up to 35 h per wk)	4 (19)	
	Unemployed and currently looking for work	1 (5)	
	Student	8 (38)	
	Stay-at-home parent	1 (5)	

^a^Participants were allowed to select >1 answer for the education question; thus, the total percentage may be >100%.

### Attitudes Regarding the Impact of AI-mHealth on Users’ Health and Data

#### Influence of AI on Health Awareness and Management

Participants expressed positive sentiment toward the potential for AI-mHealth apps to support health awareness, health maintenance, and preventative care. Several users acknowledged the value of AI-mHealth in encouraging users to take steps to manage their own health, with one participant noting that “Sometimes we can’t acknowledge the moment ourselves” (P101) and another noting that a major benefit of the incorporation of AI into mHealth is its ability to “[help] you know when you should be thinking about your mental health and wellbeing” (P130).

While improved health awareness was perceived as a benefit of AI-mHealth apps, participants also described ways in which they felt such technology could undesirably influence their health behavior. While some participants referenced having “blind trust” (P096 and P117) in AI-mHealth to manage aspects of their health, others emphasized that AI-mHealth apps should act as a supplement to an individual’s health management, with one user noting, “Any AI use should just be a healthy sidestep to us taking control of our mental health versus taking over and trying to do it for you” (P130). Another described a desire to feel in control over the management of their own health and explained how AI-mHealth could interfere with feelings of autonomy:

Part of me doesn’t like to have too many orders. I like having the ability to choose a bit...When it comes down to health, my body and my movements, that’s one of the main things that we can control, that we should control. And someone telling me to “Do this right now,” it’s just eh. The way I view health is as an investment in myself, but I also want to do that, I want to invest in my health. I want it to be voluntary in that sense. And just having [technology do that instead] makes me feel like a robot, just not completely autonomous.

P101

#### Perceived Value of AI for Mental Versus Physical Health

When discussing their attitudes toward AI-mHealth apps, users differentiated between the domains of mental health and physical health. Multiple users noted that physical health could be more easily quantified than mental health, thus making mental health more complex and difficult to measure. Users commented that “Physical health is more quantitative and mental health is more qualitative...there is so much more to unpack from it than just a number for physical health” (P101); “Mental health is, more often than not, harder to measure than physical health” (P104); and “Technology can compensate for physical health, as opposed to, mental health can become so complicated” (P130). Several users noted that, because of mental health’s resistance to measurement and quantification, they would be more skeptical of AI-mHealth recommendations for mental health and more accepting of recommendations for physical health:

[AI] could work for certain things but...It maybe needs more work on other aspects, especially when it comes to emotions and people’s decisions. In math, it totally works. But if it can tell you when you’re going to have this episode of sadness or happiness, I’m not very sure about that.

P118

Physical health has been a lot more black and white. When I get a suggestion for my physical health, I don’t question it as much. I am more likely to accept it...I am less hesitant about software that is telling me to do a physical activity.

P101

Users further emphasized that they would desire the ability for greater personalization, customization, or control of AI-mHealth apps that target mental health, with the aforementioned user noting the following:

With the mental health app, I want it to be more customizable. I feel like it requires more factors to consider before suggesting or working.

P101

#### Acquiescence Regarding Data Collection

Users expressed a reluctant acceptance toward the amount and type of personal data collection that occurred while using AI-mHealth apps. Although many described discomforts regarding the volume of data collection, they acknowledged that it felt “unavoidable” due to how deeply ingrained this process is in modern technology use (Textbox 1).

**User quotes regarding data collection.**

- “[Sharing data] feels unavoidable to me unfortunately. I don’t feel great about this but it feels like there’s some of my data that I will just have to give up.” [P097]
- “I feel like our generation is so used to using applications and we don’t really question what happens with [our data].” [P101]
- “Ideally I don’t want that to happen, but I think in reality they already have access to all of our data.” [P105]
- “I am very into providing my data for research purposes, because I know that [data collection] is still happening anyway.” [P106]
- “I already know companies use my data to sell, but that’s just also inevitable.” [P114]
- “I honestly just think it’s so much a part of everyday life. I would not say I am 100% comfortable with all the data collection that is happening.” [P124]
- “I don’t particularly want data that isn’t already out there to be going out there...I deeply hate that expectation of handing over [my] data about what I am doing.” [P131]

Several users admitted a conflict between their theoretical preferences relating to data collection and sharing and their actual use of technology, with one user summarizing the following:

I feel like in theory I care a lot about how my data is being used, what type of data, who it’s being sold to. In practice, I feel like I don’t really know what kind of data [the apps I use] collect.

P097

A different participant acknowledged that, while data sharing likely would not incur any real risks, their negative feelings toward these practices were based “on principle” and their desire to feel as if they had control over information about themselves:

When I think about data privacy, it comes more down to the principle of wanting my information to stay private and not feel like I am being tracked all the time. Because in reality when I think about it, nothing terrible seems like it is going to happen if my information is going to be used other than them telling me what to buy. It is not a huge issue, but it is almost the principle of the thing, and wanting to feel like you have ownership over your day-to-day and it not being up to outside controlling factors.

P130

### Influences on User Trust in AI-mHealth

#### Physician or Expert Recommendations

Multiple users agreed that endorsements from physicians or guidance from health or regulatory organizations would increase their trust in an AI-mHealth app and their willingness to use it, with one stating that “having some kind of affiliation with an actual medical professional does actually instill a little more trust and make you more willing to share your information” (P130). Several users specifically mentioned that they would be more comfortable sharing personal health data with apps that were recommended to them by a trusted physician. One user described the following:

Depending on the original intent of the app, if it greatly benefits my health, if my therapist told me to use this specific app and then I saw that it had great improvements in my health because it tracked my specific triggers or mental health and it took record of everything, I don’t think I’d question the data that is being collected.

P114

One user specifically referenced the Food and Drug Administration approval of AI-mHealth apps, commenting on how the regulatory process of Food and Drug Administration approval gave them confidence and had the potential to give other users more trust about using such apps:

One of the applications I used is a FDA approved method for tracking something. That gives me a lot more trust. For AI situations where it is making recommendations based on your health, it should be like any other thing. Drugs that increase your mental wellbeing, those are FDA approved. It should go through the same scrutinizing process and the same regulatory process as well. I think the public would be more willing to use it too.

P096

#### Attitudes Toward Technology Companies

Users expressed mixed levels of trust in companies that develop AI-mHealth apps. Several users saw the reputation of large companies as a reason to have trust in being a consumer there because these companies were “reputable and reliable” (P114). Two participants specifically referenced feeling more comfortable sharing their data with larger, established companies, noting that “I would feel safe...if it’s a reliable company like Google” (P127) and “A big company that people would know, I would feel comfortable sharing the information” (P130). Although most users did not provide additional details about why they trusted larger companies, one participant acknowledged the advantages that large companies have that may keep user data safer:

The paradoxical part is I’d rather almost want it to be Amazon other than somebody I don’t know, because I know that Amazon is large enough and asks the employees to do a better job at keeping my data safe from outside people.

P131

Others described mistrust in technology companies based on their perceptions about how these companies collect, use, distribute, and profit from users’ data, with one user stating the following:

They use all this data to benefit their company and they have all this money, but none of the users receive it...I feel like they are just using [our data] for personal selfish gain without it really benefiting anybody except their own company.

P114

Another user cited the history of such companies benefiting from the collection of personal user data as evidence against trusting them to genuinely work toward creating benefit for their consumers:

All the mental health applications, we can see that they want to earn money. I think the consumers that are looking for mental health services in any kind of different formats, including applications, are essentially looking for a little more authenticity in their services because it is mental health care. If the consumers can see they profit over us, once they see that, it’s like, “I don’t really trust this service.”

P106

#### Reasonable but Not Necessarily Explainable Output

Multiple users indicated that understanding the rationale behind an AI-mHealth app’s recommendation was not crucial to their trust in the app. Users acknowledged the complexity and black box nature of AI reasoning and did not think that having increased access to weights or other data used by the AI would be beneficial to their own trust or understanding. Instead, there was agreement that the perceived reasonableness of an AI recommendation had a greater influence on their trust and willingness to implement a recommendation (Textbox 2).

**User quotes regarding the explainability of artificial intelligence output.**

- “I can appreciate the fact that a complex algorithm is a black box, but as long as I know that their training sample was diverse and big enough and their results are interpretable and make sense I don’t mind as much. I think it’s fine that they don’t know what is happening.” [P97]
- “I’ve learned that the neural network is a black box. You can only know what you add in and what you get out of it. It’s still a mystery to everybody. I have accepted that with the AI tools that I have used in the past. If the conclusion isn’t outrageous and I think it’s useful, I’ll do it.” [P104]
- “I believe the rationale for the AI is based on a bunch of different factors and I don’t think I’m interested in learning which factors weigh a little bit more than the other.” [P105]
- “It’s the zeros and ones in the computer. How can I interpret that and equate that with the human thought process? It’s not possible.” [P124]
- “I honestly don’t care as long as the actions and recommendations don’t seem ridiculous, I would not care about the exact reasoning.” [P126]

In addition, several users conveyed tolerance for the “black box” limitations of AI recommendations, noting that humans often cannot explain exactly what drives their intuitions and judgments, and extending this idea to AI:

There are some things that we think about and we can’t really explain our reasons for it...We don’t really know how to quantify certain things, so I can’t completely blame the AI for having a black box concerning my data either.

P114

Multiple other users similarly compared the limits of explainable AI to the covert processes of the human brain:

It is sometimes really hard for even the software engineers to design the specific algorithm to know exactly what is going on in that thought process. It is really hard for even us to walk someone through, “I thought about this and then this thought led to that, because of the specific factors...” It is really hard even for ourselves to talk that through.

P106

[Humans] can’t always pinpoint exactly where something comes from, where an idea comes from, where feelings come from. If I’m being empathetic to the AI, I would be like, “Yeah I get that. You don’t know where this suggestion is coming from but you feel like it’s correct.” I would probably trust that.

P115

### Preferences Related to Information Sharing in AI-mHealth

#### Type of Data Being Collected and Used

Several participants clarified that, while they felt comfortable with not knowing *how* an AI app makes a decision, it was important for them to understand *what personal* data were being collected and used in such decisions, as described by these participants in the following statements:

The initial information they are collecting I would want to know. How those decisions are made, I definitely can understand that that would be too complicated to be explained. That I don’t really mind.

P130

It wouldn’t be too much of an issue if they can’t explain how it’s making the decision, but at least knowing what is being considered in the process would give me some peace of mind.

P101

One of these users wanted to know, “What information do they need from me to decide?” (P130), while other users summarized, “I would love to know what kind of data is being taken in” (P127) and “I would really appreciate knowing what behaviors and responses they are using to create that output...I think people 100% deserve to know every piece of data they are using and knowing what that is for” (P097). Several others described how being provided with information regarding the type of personal data that are being collected and used in an algorithm’s decisions would increase their willingness to consistently use an app (P096, P117, and P120).

#### Future Uses of Personal Data

Many users expressed a desire to have access to information regarding the potential future uses of data collected via their use of AI-mHealth apps. Specifically, they wanted to know if personal data collected by an app would be sold to or shared with other corporations or entities (Textbox 3).

**User quotes regarding future uses of personal data.**

- “I would like to know if the applications that I’m sharing data with have plans to sell that data.” [P097]
- “[I want to know] what it’s being used for. And I think it should only be used for the optimization and my de-identified data should be used for the training of the model to make it more accurate, better for future uses.” [P104]
- “I really would like to know what data are being collected and how exactly they are being used. Because I don’t want my health data to be floating around.” [P106]
- “I would love to know who is the end user of my data. Has my data been sold to another person?” [P111]
- “I really want to know specifically how they use it. Are they selling my data, what agencies or companies have access to that? What is the background of these companies? Are they just going to spam email me, put it on the dark web? I really want to know the security of that.” [P114]
- “How are you using my data, is it going anywhere where it’s identifiable?” [P120]
- “We are hearing stories that they are selling data. I would love to know if the data that is collected, is it being sold to hospitals or is it being sold to the black market? If it is sold to a hospital for further research then I will be okay with it, but if the app is using it for other malicious activities, that would be very wrong.” [P127]

Multiple users discussed how the collection of highly sensitive health data increased their level of concern about potential sharing or selling of the data to third parties and their desire to know whether this was occurring. Three users specifically mentioned menstruation-tracking apps as examples of health apps where concerns of data being shared or sold involved additional or elevated risks (P097, P114, and P115). One participant argued that exposing sensitive health data to third parties could lead to serious social, legal, and medical harms:

[I would want to know] who has access to that data. I read some discourse about the banning of abortion and then they were telling people to delete period tracking applications because if they’re able to detect when you’re on your period or trying to get an abortion, you could legally be arrested for that...If [the company is] able to sell that data or give the government access to that, it could potentially put certain people in danger.

P114

#### Accessibility of Information

Users expressed a range of suggestions about how companies might better communicate to users about the collection and use of their personal data. Multiple users described the lack of engagement that users have with traditional terms and conditions pages, noting that “Many people just don’t read them, and I am one of those people usually” (P115); “For me, and probably a lot of people, [when] reading those small text notices, I always push ‘agree’ and move on” (P096); and “A lot of people blow through that stuff and are like, ‘Yes, yes, accept’” (P120). The length and complexity of these agreements were frustrating to users, with one commenting that “I just don’t like how a lot of us aren’t really informed how our data is being used, and then they make it so complicated to the point where we don’t really look through it” (P114).

Several users described wanting to receive clear and digestible information regarding the risks and benefits of AI-mHealth apps as a more straightforward way to understand considerations related to their data. Two users described that, “It would be good to know the risks and benefits” (P096) and “[I would like] an explanation of pros and cons, rather than a sales pitch-type view of it” (P124). Other participants commented that companies should provide a “condensed version” of the terms and service agreement that “just gives the bullet points” (P101), with one participant noting how a shift toward providing more accessible information could demonstrate a company’s concern for and efforts to safeguard users:

Privacy policies are often not very accessible. I want to see, not just do you provide the information, but do you provide it in a way that is clear with its language which to me also expresses that you care about what you are doing with the data? [To see] if you take the time and energy to make something that people understand because you care about conveying that information.

P131

One participant further expanded on their request for more accessible information regarding the risks, benefits, and data policies of an AI-mHealth app and posed an analogy to the informed consent process they completed for this interview study:

I want the app to highlight the very important data that they will be collecting and to put that into layman’s terms so that I can just understand it very easily...I am thinking of something like the informed consent procedure that you just did to me. The document itself looked pretty intimidating...[but] you highlighted the components I really needed to know as a study participant...If the app could do something like that...if it can be that transparent and simple, that will definitely boost my trust and credibility as the app’s consumer.

P106

Some participants suggested technology- and media-based alternatives to the current text-based terms of service agreements that they felt would provide information relating to risks, benefits, and how personal data are collected and used in a more digestible and accessible format. Two suggested videos as an alternative to text (P096 and P115), with one noting that this format could be useful for “forcing” users to engage with the information:

If it were presented in a really digestible way where you were basically forced to read it or hear it if you use the app, like a video where you couldn’t move on unless you play the whole thing.

P096

Multiple other participants commented that apps could have associated websites where users could look up information if questions arose through their use of the app, with one noting the following:

It would be great to have a resource that is just live all the time. A website where you can access that information and then contact them if you have questions that aren’t answered on their website.

P120

## Discussion

### Principal Findings

In this semistructured interview study, users expressed attitudes and concerns about a number of ethical considerations related to the use of AI-mHealth apps, including considerations related to autonomy, privacy, trust, transparency, and information sharing. The major findings are discussed in detail in the subsequent sections.

#### The Value of AI-mHealth: Perceived Limitations of Apps for Mental Health

Interviewees expressed comfort with the idea of using AI-mHealth apps to supplement their health management, citing their value in organizing and tracking health data and providing users with prompts or knowledge that could benefit them. Several users were adamant that AI-mHealth should supplement rather than replace health care services and preferred technologies that encouraged informed and autonomous health decision-making, as opposed to more assertive recommendations, which they felt could be perceived as orders that could ultimately detract from health awareness. This finding aligns with previous research by Almourad et al [27], which found that users’ feelings of increased self-awareness and a sense of control over the use were acceptance factors in their use of mHealth technologies.

Users in this study notably distinguished between the potential value of AI-mHealth apps designed for physical health and those intended for mental health. Strikingly, some were less confident that AI could be as effective for mental health use cases compared to physical health use cases. They perceived mental experience as highly personal, intimate, and resistant to observation and measurement (admitting also the limits of human introspection) and therefore did not feel that AI-mHealth apps could “know” them well enough to make accurate inferences that lead to helpful predictions or guidance. These attitudes deviate from previous findings among avid users of AI mental health chatbots, who report high engagement and trust in such apps [28], and suggest that less experienced or laypersons, such as those in our study, may be more hesitant to accept predictions and guidance that rely on AI inferences regarding more subjective and phenomenal mental processes (eg, underlying emotions or affective states) compared with apps that target physical health. The design and implementation of AI use cases for mental health will benefit from additional empirical inquiry to help understand how users with less experience think AI can benefit this domain and develop a clearer picture of ethically salient factors that may influence adoption.

#### The Collection and Sharing of User Data: Acquiescence and Mistrust

There was a general admission among interviewees that, as users of modern technology, they often must concede control over their data and their future uses. They acknowledged discomfort with the fact that personal data are systematically collected through their devices, partly to help advance corporate and business interests, but expressed acquiescence about their ability to prevent or change this reality. These attitudes have been documented frequently in the technology literature, having been described as privacy cynicism [29], digital resignation [30], privacy apathy [31], and privacy fatigue [32]. Notably, participants in our study were young adults whose interactions with technology have likely always involved concessions relating to privacy, possibly resulting in a reluctant acceptance that the use of technology by default incurs the loss of certain privacies. Future studies assessing feelings of acquiescence across technology users of different age groups would be of interest.

In addition, participants in this study described how the pervasiveness of data collection and sharing influenced their trust not just with specific technologies but also with the corporations or entities associated with them. Some expressed greater trust in large companies that they believed were better suited to protect their data, while others asserted no trust in these same companies due to their historical collection of user data. This, too, is consistent with findings from the technology literature, which found mistrust in corporations that users associate with data sharing. As was described by Lutz et al [33] in their survey study, the potential benefits of an individual technology or app may be trumped by “mistrust and powerlessness in relation to those platforms ostensibly providing the infrastructure.” Although a recent user-review analysis found an overall high degree of public trust in individual AI-mHealth apps for mental health, our results, along with findings from the technology literature, indicate that there are users whose attitudes toward AI-mHealth are influenced by their existing trust, or lack thereof, in large technology companies [34]. More research is needed to assess how the systematic collection and distribution of personal data have contributed to users’ trust or mistrust in technology companies and the impact that this may have on their trust in and adherence to AI-mHealth apps.

#### Users’ Desire for Information: Understanding What, Not How

In a previous interview study with users of AI-mHealth apps representing an array of mHealth use cases, participants reported desiring more straightforward and precise information about the apps, including how they used AI, how the AI-enabled features worked, and how well the AI might solve the health needs the apps purported to address [35]. Participants in this study consistently expressed similar high-level concerns; however, they were less concerned with *how* an AI-mHealth app generated its outputs than they were about *what* types of data it collected and used to generate its feedback or guidance. A related, frequently expressed worry was whether their personal data could be used by additional parties or for other purposes in the future. These findings suggest that AI-mHealth users may put less stake in understanding the underlying technology and reasoning procedures driving an app and have more reservations about the collection and use of their personal data.

Experimental studies with users of AI tools in health care and other sectors engaging in AI innovation have shown that enhanced explainability is not always favorable to user understanding, decision-making, or trust [36-38]. Our results contribute much-needed stakeholder data to this literature and reveal that explainability tools or technical explanations of algorithms and models may not be crucial to secure users’ trust and facilitate adoption, provided that they have access to resources that assuage personal data–related concerns.

Beyond noting that understanding an AI’s reasoning procedure was not essential to their sense of trust, several participants in this study strikingly related black box algorithms to human intelligence and decision-making processes. Specifically, they described how both algorithmic and human reasoning involve processes that are obscured from the observer. A previous interview study by Gkinko and Elbanna [39] documented the tendency of people to extend empathy to AI chatbots into their workplace environments; however, the association of black box AI decision-making and human intelligence appears to be a novel finding that has not been previously documented among users of AI-mHealth apps. This association provides a reminder that humans are accustomed to making decisions based on mental processes that are not necessarily able to be articulated or explained. Taking action based on incomplete or obscured information is not a novel experience for humans, and as our participants acknowledged, this willingness could possibly extend to their interactions with AI.

#### Informing Users: Concerns and Recommendations

Users in this study repeatedly acknowledged that they do not read terms and conditions agreements, which they felt were overly lengthy and not designed to inform users. Participants offered a range of alternatives for how they would prefer to be informed about the use and future distribution of their personal data. User-friendly summaries of risks and benefits of an app were suggested, as were videos or accessible websites. As highlighted by several participants, the inclusion of easily digestible lists or videos regarding risks and benefits of use is a fairly minor addition to an app that could be incorporated before or after existing terms and conditions pages. Comments from our participants indicate support for concepts, such as the “Nutrition Label for Privacy” proposed by Kelley et al [40]. As AI-enabled technology that collects increasing amounts of user data becomes more accessible to the public, offering users clear and comprehensible information regarding the use and sharing of their data may influence their trust in a tool and willingness to incorporate its recommendations.

When discussing how information sharing in digital health could be improved, one participant referenced the informed consent method that is typically used in medicine and research. Informed consent forms share similar challenges to terms and conditions documents, in that they often include content that is not necessarily relevant to participant understanding or decision-making, but that instead is required for the purpose of protecting the interests of the funding or managing institutions. Studies testing shorter consent forms have found that participants were more likely to fully read shorter forms and that those exposed to shorter forms comprehended more of the information, indicating that the length of the terms and conditions pages could be a primary factor in users’ tendency to bypass them without engagement [41]. Furthermore, the accompanying informed consent process in which the information from informed consent forms is distilled into accessible language that highlights the risks and benefits is often positively rated by participants, possibly indicating that terms and conditions pages that are followed by summaries highlighting the information relating to specific risks and benefits may be well-received by users of AI-mHealth apps [42,43]. Informed consent processes are established within medical practice and research and could provide a useful road map for AI-mHealth apps, which may require both templated legal disclosures and access to product information that encourages participant engagement and understanding.

### Limitations

This study has several limitations relating to its sample. Most notably, all participants were aged between 20 and 36 years at the time of the interview. It is documented that attitudes toward technology are influenced by age and life stage, so the findings from this study should therefore not be generalized to older populations [44,45]. Furthermore, our sample primarily consisted of individuals who identified as female and belonged to a racial or ethnic minority group. As we did not specifically intend to prioritize the interviewing of these populations, the attitudes documented in the interviews and analysis should not be assumed to be representative of all users of mHealth apps. In addition, our findings are limited by the fact that interviews were completed in 2022, before the first release of ChatGPT, which represented a major shift in the public’s perceptions toward AI and AI-enabled technologies.

### Conclusions

In this qualitative study using semistructured interviews, users expressed their attitudes and concerns about a number of ethical considerations in the use of AI-mHealth apps, including considerations related to autonomy, privacy, trust, transparency, and information sharing. Notably, users distinguished between the potential value of mental and physical AI-mHealth apps, cited existing feelings of loss of control and ownership of their data in everyday use of technology that appeared to influence their trust in AI-mHealth broadly, and expressed a desire for more proactive and accessible information sharing about aspects of AI-mHealth apps. These findings present recommendations for consideration in the development and distribution of AI-mHealth apps that may enable greater ethical alignment between producers and consumers of AI-mHealth apps. Future evidence-based research examining the intersections between mHealth and AI with the attitudes of relevant stakeholders is still needed as is research that analyzes how the actual use of AI-mHealth apps aligns with the ethical perspectives identified in this study.

## Data Availability

The datasets generated or analyzed during this study are not publicly available in order to protect the identities of participants but are available from the corresponding author on reasonable request.

## Abbreviations

AI: artificial intelligence
AI-mHealth: artificial intelligence–enabled mobile health
mHealth: mobile health
